# Lessons from community engagement to improve COVID-19 diagnosis and treatment in Cochabamba, Bolivia

**DOI:** 10.1080/16549716.2024.2358602

**Published:** 2024-06-11

**Authors:** Elizabeth Posada, Nilce Mendoza, Cristina Alonso-Vega, Claire Billot, Beatriz Mallén Muñoz, Leonardo de la Torre, Adalid Paiva, Luis Villarroel, Regina Rabinovich

**Affiliations:** a Barcelona Institute for Global Health (ISGlobal), Barcelona, Spain; b Fundación Ciencia y Estudios Aplicados para el Desarrollo en Salud y Medio Ambiente (CEADES Foundation), Cochabamba, Bolivia; c Consejo Social Municipal de Salud Cochabamba (CSMSC), Cochabamba, Bolivia; d Quatrim, Cochabamba, Bolivia; e Harvard TH Chan School of Public Health, Boston, USA

**Keywords:** COVID-19, community engagement, Bolivia, co-creation, health promotion

## Abstract

**Background:**

Community engagement is recognized as a vital component of health-related research and programs, particularly during infectious disease outbreaks and epidemics. Despite the importance of engaging communities in the response to COVID-19, relatively little research has examined how this was (or was not) achieved, and even less in low- and middle-income countries. This article describes the community engagement that accompanied efforts to strengthen COVID-19 diagnosis and treatment as part of the ECO Project in Cochabamba, Bolivia and highlights lessons for future pandemic response.

**Methods:**

Community engagement involved formative assessment, co-creation to develop a health information campaign, ongoing community listening and evaluation. Qualitative data were collected during workshops, project meetings and focus groups. Questionnaire-based surveys were conducted to assess COVID-19-related attitudes, knowledge and practices.

**Results:**

The collected data highlighted the value of working closely with well-established community health committees and involving community members with social media skills in the design of COVID-19-related messages to address on- and offline misinformation. Co-creation sessions enabled the adjustment of the information campaign in terms of content and approach based on the needs and preferences of community members and health staff. The continuous listening with community and health personnel facilitated the ongoing adaptation of project activities.

**Conclusion:**

Through a stepped and multi-pronged approach, incorporating co-creation and community listening, the engagement could respond to emerging local challenges during the pandemic. The project created spaces for dialogue and opportunities for collaboration that strengthened links between the community and the health services.

## Paper context

**Main findings:**

Key elements of community engagement to improve COVID-19 diagnosis and treatment in Cochabamba, Bolivia, included working closely with well-established community health committees, involving community members with social media skills in the co-design of COVID-19-related messages, and continuous listening with community and health personnel facilitated the ongoing adaptation of project activities.

**Added knowledge:**

With little research on community engagement for COVID-19 diagnosis and treatment in Latin America, this study reports the results of mixed methods research on the impact of a comprehensive approach to engagement that highlights lessons for future health emergencies.

**Global health impact for policy and action:**

Lessons for engagement in health emergencies include the need for a multi-pronged approach, incorporating co-creation and community listening, to respond to emerging local challenges.

## Background

Community engagement is recognized as an essential element of health-related research and interventions [1,2]. Despite some broad definitions, it can be a slippery concept and subject to varied interpretations across disciplines and between funders [3,4]. It often becomes a catch-all term for diverse sets of activities, which include informing, consulting, collaborating and/or sharing leadership with people who are the targets of – or who have a stake in – health-related research or intervention programs [5,6]. The overall aims of community engagement can also diverge, with some placing emphasis on the instrumental benefits of engagement activities, for example, in terms of increasing uptake of interventions or study participation, whereas others point out the ethical and moral dimensions, to redress power imbalances and ensure that research, interventions and/or programs are responsive to the needs and priorities of those that have been historically neglected and exploited, particularly in the context of initiatives led by actors from higher-income groups or countries [1,7].

During health emergencies, such as outbreaks of infectious disease, the potential benefits and pitfalls of community engagement become particularly pronounced. This lesson had supposedly been learnt from health crises in the late 20th and early 21st century – such as outbreaks of Ebola, MERS etc [8]. However, in many settings, the response to the COVID-19 pandemic, particularly during the early phases, suggested otherwise [9]. In a background of uncertainty about transmission mechanisms, risk factors, mortality rates and long-term health impacts, the responses from public health authorities and governments more broadly were mixed, sometimes with little meaningful community engagement [10]. Particularly, at the outset, efforts to engage communities were commonly superficial – focused on top-down information provision to the wider population – and sometimes completely absent [11,12]. Given the complex information environment during COVID-19, this combination likely hindered the potentially positive effects of the responses [11].

In Latin America, there is a tradition of community participation (or mobilization) in health (and economic development) programs [13]. Vector control programs, especially those addressing dengue transmission, have emphasized community involvement to increase their effectiveness [14]. The outbreaks of Zika virus prompted efforts at community involvement in the design of responses, particularly vector control, with some encompassing a participatory planning process that included community leaders and residents [15]. For the control of Chagas disease in Latin America, some projects have demonstrated the value of community participation [16]. Nonetheless, for Chagas disease programs, in which household vector surveillance is essential to identify the presence of vectors in households, meaningful community engagement has been often absent to the detriment of vertical programs [17].

As a vital aspect of the response to public health threats, to prepare for future pandemics, efforts to understand the success and failures of strategies to engage communities during the COVID-19 pandemic response are crucial [18]. A key element in building evidence around community engagement in response to health crises is a detailed account of its aims and activities, as well as the challenges faced and overcome [19]. To this end, this article describes the community engagement that was undertaken as part of the ECO project (Enhanced and equitable coverage of COVID-19 testing and treatment in Bolivia and Paraguay), a UNITAID-supported multi-disciplinary response to the COVID-19 pandemic in two countries in Latin America. This article focuses on the activities undertaken in Cochabamba, Bolivia, with a view to learning lessons that are useful for community engagement practitioners addressing (future) health crises in Latin America and beyond.

## Methods and setting

This article draws on a mixed-methods evaluation of community engagement undertaken as part of the ECO project in Cochabamba, Bolivia. The mixed methods design included qualitative research methods (focus groups and observations), which provide a description of the processes of community engagement, the challenges faced and overcome, from the perspective of multiple stakeholder groups (project staff, healthcare providers and the wider community) and more quantitative questionnaire-based surveys to examine perceptions of the community engagement (among healthcare providers and wider community) and its effect on COVID-19-related knowledge and practices.

### Setting

#### Geographical and social context

**Figure 1.**
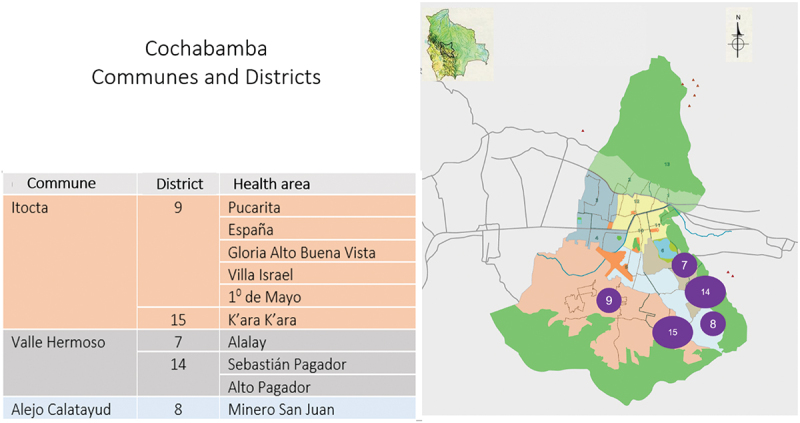
Map of “Zona Sur”, Cochabamba.

In Bolivia, Cochabamba’s ‘Zona Sur’ was selected for the ECO project with the assistance of the municipal authorities, based on the identified need for improved diagnosis and treatment of COVID-19 in the area. Zona Sur is an area comprised of three communes: Itocta, Alejo Calatayud and Valle Hermoso, with estimated population of around 300 000 residents [20] (Figure 1). The area is characterized by in-migration from rural areas, which has led to recent population increases. The economy is dominated by informal commercial activities. The social structures present in rural areas maintain their significance in everyday life in this more urban setting. Important social structures include local health committees, which are each connected to a primary care facility and act as a link between the surrounding community and the health center. The local health committees are coordinated by a Municipal Social and Health Council, which made up of members elected by the community.

#### Context of COVID-19 in Cochabamba

**Figure 2.**
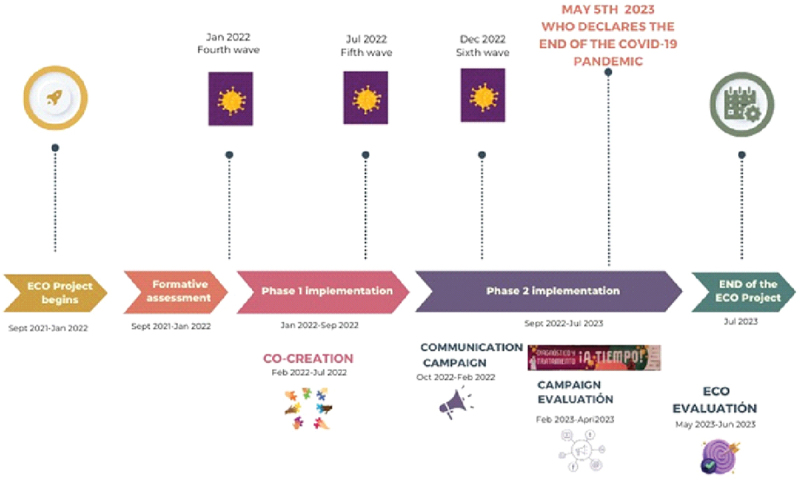
The ECO Project timeline and waves of COVID-19 infections in Cochabamba.

In the Department of Cochabamba, the first case of COVID-19 was recorded in March 2020 [21]. As in many settings, the pandemic subsequently played out with a sequence of peaks or waves of cases (generally accompanied by peaks in hospital admissions and deaths), linked to the circulation of new virus variants and/or the waning of vaccine-induced and/or acquired immunity [22]. Up to the end of 2022, six of such peaks were recorded in Cochabamba, leading to a total of 14,000 confirmed cases and more than 3000 reported death [21]. These are likely significant under-estimates, given the limited availability of diagnostic tests, particularly during the early phases of the pandemic (see Figure 2 for an overview of the various waves during 2021–2023).

#### Enhanced and equitable coverage of COVID-19 testing and treatment in Bolivia and Paraguay (ECO)

The overall aim of the project was to assist Bolivia and Paraguay in their response to COVID-19, specifically through improving diagnosis and management of COVID-19 infections. In Bolivia, the ECO project was conducted in five districts, and ten health areas, each one the catchment of a single primary care facility. The district referral hospital located in the area was also included. Each health center serves a population of between 10,000 and 20,000. The ECO project was initiated in September 2021 and activities on the ground ended in July 2023 (see Figure 2).

#### Community engagement in the ECO project

**Figure 3.**
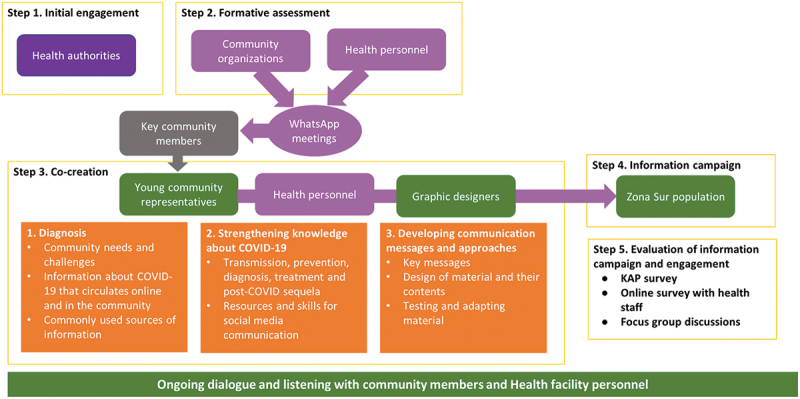
The steps of community engagement in the ECO project.

The ECO Project incorporated a community engagement component to facilitate the timely diagnosis and treatment of COVID-19. The community engagement entailed a collaborative approach to the (co)creation, implementation and evaluation of an information and education campaign with the intention of being responsive to the needs and preferences of the target communities. This required a multi-stage process [1]: first, initial engagement with stakeholders [2]; then a formative assessment, that informed the [3] co-creation sessions, which were used to develop the subsequent [4] community information campaign, which was followed by [5] collaborative evaluation in the form of a knowledge, attitudes and perceptions (KAP) survey that was administered to a random sample of community members in the target facility catchment areas (Figure 3) and an online questionnaire-based survey that was completed by health personnel at participating facilities. In addition to the questionnaire-based survey in the final stage, qualitative data were collected throughout the engagement progress, and particularly focus group discussions during the evaluation.

The engagement targeted multiple stakeholder groups: health authorities, community organizations, health personnel at selected facilities, key community members and young adults from the community. Co-creation was a priority for the engagement process and integral to the design of messages for the community education and information campaign as well as the media used in the campaign. Drawing of a collaborative process of research working with stakeholders [23], the co-creation aimed to ensure that the messages were accessible, comprehensible and addressed the information needs of the target communities. The intention was to include co-creation participants from a diverse stakeholder group, including healthcare personnel.

Each element of the ECO project, including the community engagement, was underpinned by a theory of change that linked outcomes with activities, indicators of success with their means of verification (the methods used in evaluation) and the assumptions that underpinned the achievement of the outcomes (see supplementary information). The theory of change for the community engagement was organized around three key outcomes related to the areas of engagement: the formative assessment (generate information on the behavior of the target population in relation to COVID-19 diagnosis and treatment); the co-creation and information and education campaign (strengthen knowledge attitudes and practices of the population for timely diagnosis and treatment of COVID-19) and evaluation of the ECO Project (community engagement and other) activities.

### Methods

#### Qualitative data

**Figure 4.**
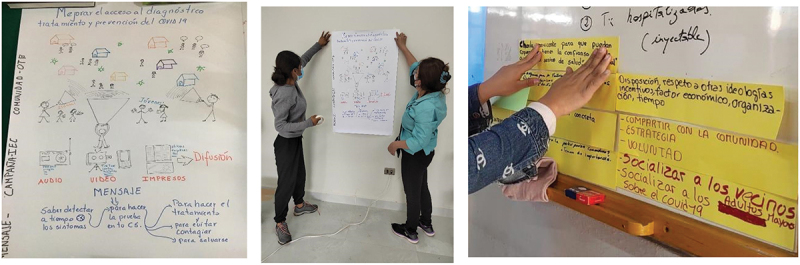
Images from co-creation sessions at the K’ara K’ara cultural Centre.

The data presented in this article are drawn from project documents, field notes, meeting minutes and photographs (for example see Figure 4) taken during the process of designing, implementing and evaluating the community engagement components of the ECO project in Zona Sur, Cochabamba. To facilitate learning lessons from the community engagement activities, project staff kept detailed notes of activities, including demographic information on participants, the materials used, the responses of participants. Throughout the implementation of the project, additional discussions, regarding progress and challenges of the project were conducted with healthcare personnel at the participating facilities.

#### Knowledge, attitudes and practices survey

To assess the impact of the community engagement on COVID-19-related knowledge, attitudes and practices (KAP), a questionnaire-based survey was undertaken. In this article, we present an overview of the results of this survey. The questionnaire-based survey was conducted face-to-face by trained community members, with a view to maximizing the response rate (and addressing concerns about the security threats that an entirely external survey team would face). All the community interviewers received training from the ECO Project team and were accompanied by ECO Project team members and community leaders, who also provided them with a list of potential respondents, selected randomly from the catchment population.

The KAP data collection tool was designed based on the intended outcomes indicators from the ECO project’s theory of change (see Table 1). The tool included 28 questions that focused on knowledge of COVID-19, attitudes to diagnosis and treatment of COVID and awareness of population groups most vulnerable to series COVID-19 disease. The tool was then piloted with community members in the catchment area of the district hospital. The tool was administered face-to-face using mobile phone-based application QuestionPro (QuestionPro Inc. Austin, Texas, USA).

**Table 1.** The theory of change for community engagement in the ECO project.

Outcomes	Indicators	Means of verification	Assumptions
Generate information on the behavior of the population in relation to the diagnosis and treatment of COVID-19	Identify the health seeking routes with regard to COVID-19 diagnosis and treatment	KAP survey	The survey protocol is validated Valid informed consent Local authorities/leaders of social organizations have facilitated access to communities
	Perceptions about barriers to accessing COVID-19 diagnosis and treatment in health facilities		
	Perceptions about facilitators to accessing COVID-19 diagnosis and treatment in health facilities		
Strengthen the knowledge, attitudes and practices of the population to achieve early diagnosis and treatment of COVID-19 (within the first 5 days) particularly in populations with risk factors	Percentage of the population aware of the importance of early diagnosis and treatment (within 5 days)		Approval and support of local health authorities, health personnel and the leaders of social organizations to carry out the information campaign
	Percentage of the population with a positive attitude toward the early diagnosis and treatment of COVID-19		
	Percentage of positive social media reactions to the information campaign	KAP survey Social media metrics Use of paper-based material in facilities	
Assess the acceptability and feasibility of a diagnostic and treatment strategy from the perspective of the community and health personnel.	Users’ level of confidence in the health facility regarding timely diagnosis and treatment of COVID-19	KAP survey Community focus groups	Diagnosis and treatment strategy implemented
	Health personnel’s level of confidence in the health facility regarding timely diagnosis and treatment of COVID-19	Survey with health personnel Health personnel focus groups	

For the KAP survey, across the catchment areas of the ten health facilities, the sample size was calculated at 594 respondents to produce confidence limits of 95% and a standard error of 0,04. Respondents were selected at random from transects based on the catchment area of each facility.

#### Online questionnaire-based survey for health personnel

In addition, healthcare personnel at all participating facilities in Zona Sur were invited to complete an online questionnaire-based survey. A non-probabilistic quota and diversity approach was taken to sampling (across all cadres at all facilities), with a sampling intensity of 15% at the referral hospital, and 25% for the 10 health areas. The questionnaire was self-administered on a tablet or mobile device via the supplied link using QuestionPro. In line with the outcomes and indicators described in the ECO project’s theory of change, the survey tool included questions on awareness of the ECO project, the activities and attitudes towards the delivery of COVID-19 diagnosis and treatment.

#### Focus group discussions

As part of the evaluation of the information and education campaign, three focus group discussions were undertaken with 28 respondents representing each of the 10 health areas: one with co-creation participants [7]; one with community members [9] from the catchment areas of participating health facilities; and one with health personnel [12] from participating health facilities. The focus groups were facilitated by members of the ECO Project (a trained communications specialist who led the ECO project in Bolivia facilitated the discussion and the project’s medical doctor took notes). The aim of the focus group discussions was to gain insights about the experiences of participating in the co-creation and the information and education campaign, their experience of the pandemic and perspectives on the preparations for future pandemics.

#### Data processing and analysis

For the KAP survey, anonymized data were uploaded daily from the tablets, checked and cleaned by the ECO Project team. The survey database was converted to SPSS to calculate descriptive statistics. For the online survey, the survey responses were uploaded to the online database. The uploaded data were cleaned by the ECO Project team and transferred to SPSS (version 27; IBM, New York, US) to calculate descriptive statistics.

During the focus group discussions, detailed notes were taken to summarize the key topics raised. The qualitative data from the project records and detailed notes were collated and organized based on the stage in community engagement process. Furthermore, in July 2023, a close-out workshop was held with project staff members. During the workshop, as part of facilitated analysis sessions, project staff reflected on the community engagement activities undertaken in Zona Sur and collectively identified the challenges faced and perceived successes of the engagement program.

## Results

Project staff described the community engagement in 60 fieldnotes, which included details of the specific activities undertaken, demographic details of the participants involved and the nature of their involvement, their responses to the engagement, the challenges that emerged and the perceived successes. This section describes the various stages of community engagement, from the initial engagement with authorities, the formative assessment, the co-creation activities, the delivery of the information and education campaign, and the evaluation of the information and education campaign. In terms of the evaluation, the results of the questionnaire-based surveys and focus groups with the community and health care staff at participating facilities are presented.

### Initial engagement with (health) authorities

In the first phase of ECO project, in October 2021, health authorities at national, department and municipal levels were invited to a virtual meeting in which the project team (based in Barcelona and Bolivia) presented the project aim and activities. Participants included representatives from the Ministry of Health, the Government of the Department of Cochabamba and the Municipality of Cochabamba. There was a positive response to the proposed project and the authorities requested an institutional agreement be made. Given the time required to finalize an agreement with the national health authorities, and because most of the work took place at a municipal level, an agreement was first signed at municipal and later at national level. With the assistance of the Secretary of Municipal Health, contact was made with Cochabamba’s Municipal Health and Social Council (Consejo Social Municipal de Salud de Cochabamba). This arm of municipal government facilitated contact with leaders in the local health system. Once this contact was established, the ECO Project team began the mapping of key health actors in the community, with a view to assessing the needs and priorities for the ECO Project’s community engagement and information and education campaign.

### Formative assessment and (continuous) community listening

As part of the formative assessment, the ECO team based in Cochabamba conducted face-to-face meetings with health personnel at the selected facilities. The objective of these meetings was to identify the needs in primary care settings and the community in terms of COVID-19 diagnosis and treatment. The formative assessment also included a desk review of COVID-19 diagnosis and treatment guidelines as well as the area’s COVID-19 epidemiological situation.

Notable health system challenges in the response to the pandemic were identified. These included insufficient equipment and human resources in primary care. There was insufficient IT infrastructure for epidemiological analysis and case follow-up. The volume of patients was high, which put health personnel under pressure and led to short, brusque interactions, and perceived low quality of care. A lack of treatment options for severe COVID-19 cases was reported. Insufficient communication between healthcare facilities and community organizations, perceived poor quality of care at facilities, and clients influenced by COVID-19 mis- and disinformation (on- and offline), were described as key barriers to the timely diagnosis and treatment of COVID-19 in the area. There was also a reported lack of COVID-19-related health information material in health facilities.

The significance of existing community organizations quickly became apparent in the formative phase. The social hierarchies that dominate rural life remain important for rural-urban migrants residing in Zona Sur. Hence, involving community leaders was quickly identified as necessary to effectively reach the wider community. The local health committees at each health care facility were particularly important as a link between the community and the health system.

In response, based on contacts lists held by local health authorities, online messenger groups comprised of community leaders were created. Because young people were identified as a key population group in terms of their use of social media and online access to COVID-related information, local health authorities, with the assistance of messenger group members, circulated an invitation to co-creation sessions for a COVID-19 information campaign.

Throughout the remainder of the project, contact with the community leaders and the healthcare staff at participating centers was maintained via the messaging groups and through visits to the participating health facilities. This community listening focused particularly on the circulating information regarding COVID-19 diagnosis and treatment, as well as prevention and overall confidence in the health facilities. This enabled the identification and response to other challenges in terms of COVID-19 diagnosis and treatment.

### Co-creation of the information and education campaign

Through online messenger campaigns, a group of 66 young people from Zona Sur expressed interest in participating in co-creation sessions. The campaign specifically sought young people from the catchment areas of all the health facilities participating in the ECO Project, who showed promising communication and social media skills, and who expressed interest in developing these competencies.

The co-creation sessions focused on gaining further insight into the COVID-19-related information that was circulating in the communities of Zona Sur and online, and the mapping of online platforms used to share information. In the first session, participants were asked to give their opinions about how to effectively engage members of the community. They emphasized the need to give information clearly, yet also spark people’s interest and to respond to their needs. They highlighted the need to make use of social networks and respect the existing social structures, such as the local health committees. They identified several additional facilitating factors, including the need to cover the expenses of those participating in activities given the economic vulnerability of many residents in the area) and ensure that the timings and location of the activities fit with the availability of the target groups.

During the series of eight co-creation sessions, activities included: offering clear and accurate information about COVID-19 transmission, vaccination and treatment, in response to the community’s frequently asked questions; developing the key messages for the communication campaign (see Table 2) and designing the campaign’s approach; training participants in social media skills, including audio and visual production. The meetings were held in community spaces and the group was divided into two working groups to focus on different elements of the communication campaign and the training, according to their preferences. A database was created to compile COVID-19-related ideas that were circulating in the community. The compiled messages were then discussed with the community leaders and the local health authorities.

**Table 2.** Summarizes the elements of the health information campaign developed by participants from the various health areas in the co-creation sessions.

Health areas	Format	Topic	Key messages
Alalay	One video	Trust in your health center and the infection control measures	Images of the infection control measures in the health center during patient care.
	Four videos		Diagnosis and treatment within 5 days of symptoms Diagnosis and treatment for at-risk groups COVID-19 has not disappeared Addressing circulating mis- information around COVID-19
	Two raps	Let’s learn to live with COVID	COVID-19 has not disappeared Timely diagnosis and treatment
Alto Pagador	A short video	Timely diagnosis to avoid COVID-19-related complications	Comparison between early diagnosis and treatment and late-diagnosis leading to critical care Importance of maintaining one’s health
España	Three YouTube videos	Prevention and risk	Prevention targeting at-risk groups COVID-19 has not left, continue with prevention measures COVID-19 exists
	Three YouTube videos	Trust in the health center	Promote community participation Encourage health personnel Build trust and continue to use face masks At-risk groups
	Video - animation	Post-COVID symptoms and at-risk groups	Post-COVID-19 health impacts on people’s everyday lives Prevention as key to prevent post-COVID Early treatment at health facilities to prevent post-COVID symptoms
	Printed comic strip	Post-COVID symptoms	Post-COVID-19 health impacts on people’s everyday lives
Gloria, K’ara K’ara and Pukarita	Three short videos	Diagnosis and treatment of COVID-19 in the health center	Motivation to seek care in the health center
K’ara k’ara	Three posters	COVID-19 has not left us and need for continued prevention	Promoting hand washing Importance of accessing health facilities
Mineros San Juan	Radio drama (Spanish and Quechua language)	The health center as key to overcoming COVID-19	Treatment seeking of a taxi-driver who is skeptical of COVID-19 but avoids complications when he becomes ill because his wife takes him to the health center
	Five radio advertisements		Key messages from the radio drama above
Sebastián Pagador	Video short story	Timely diagnosis	Story of an adolescent who recall his family’s experience of COVID-19
	Information leaflet	Basic COVID-19-related information	COVID-19 transmission and symptoms Importance of seeking care at the health facility
Villa Israel	One video	Biosecurity measures – use of alcohol	How did we prevent infectious disease transmission in the past? How do we do it now?
	One poster	Protecting persons at risk of COVID-19	The value value of early diagnosis of those at greatest risk of COVID-19 Importance of timely diagnosis to reduce complications

When asked about the ideas about COVID-19 circulating the community, the participants raised a variety of issues, many of which were present on- and offline: that the disease does not exist; that it was developed in a laboratory with the aim of reducing the global population (or specifically the elderly population in China); that it was created by pharmaceutical companies or the Chinese; that the vaccine contains a micro-chip with behavioral control properties or that the vaccine were harmful or ineffective. In terms of the vaccination programs, they also heard doubts about whether the children should be vaccinated, the need for other preventive measures post-vaccination and the rejection of mandatory vaccination. Participants highlighted social media as promulgating disinformation, but also indicated that much information was spread person-to-person. They indicated that the origins of much of this information was unclear. They labelled many of these ideas as misinformation yet pointed out that there were sources of reliable information, particularly radio broadcasts.

In the co-creation sessions, efforts were made to strengthen the participants’ understanding of COVID-19, its transmission, diagnosis, treatment, and potential sequelae. The sessions were led by ECO staff or external facilitators with extensive experience of community participation in public health programs, and health care staff from the participating health centers. In recognition of their efforts, the co-creation participants received a certificate of participation.

### The community information and education campaign

**Figure 5.**
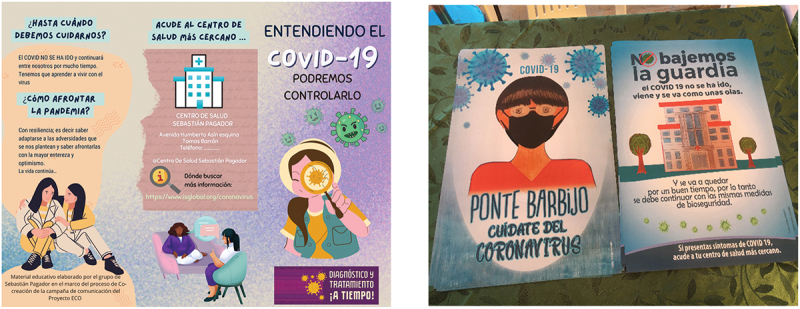
ECO project Co-created leaflets.

**Figure 6.**
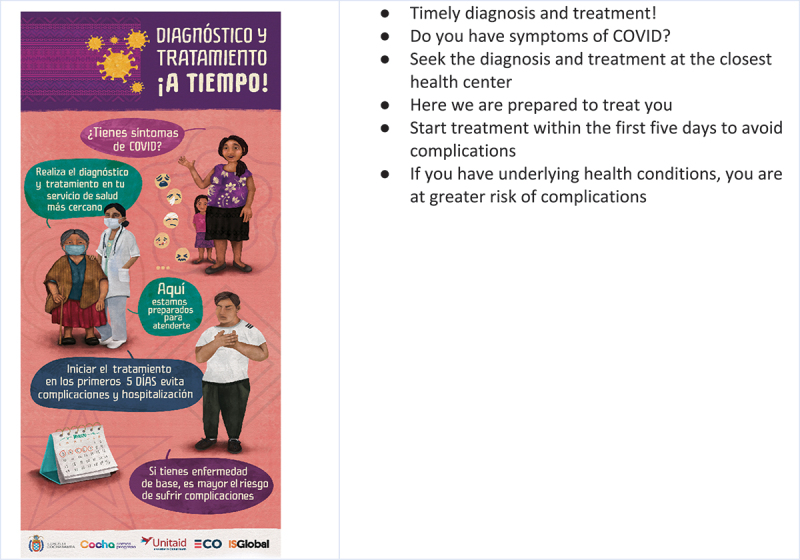
A banner displayed in participating health facilities with the key messages of the information and education campaign.

Based on the co-creation sessions, a community information campaign was designed. The campaign included online and offline components, with online elements focused on the social media tools identified in the co-creation sessions. The formats and messages varied across the health centers. Across the health facilities, the campaign included 16 short online videos (disseminated via YouTube) a printed comic strip, one leaflet, a radio drama, four short radio jingles, a rap broadcast on the radio and four posters. In addition, talks were given at health facilities in the waiting areas (where banner, flipchart, posters, and other printed materials were placed). The ECO Project’s communication team were involved in finalizing the design of the posters and other printed materials (see Figure 5). They took their cues from the co-creation participants, for example, for the figures used in the designs, which were based on images of community members provided by participants. The messages were based on the co-creation. The central message of the campaign was the timely diagnosis and treatment of COVID-19 (see Figure 6).

### Monitoring and evaluation of the information and education campaign

Drawing on quantitative and qualitative data, the information and education campaign was monitored and evaluated by the ECO Project team, in collaboration with community members. This included collecting data on the online audience for the platforms.

#### Online evaluation

In terms of the online information and education campaign, in Bolivia, the main platforms were Facebook, WhatsApp and YouTube. Links to the page were placed on the website of Cochabamba’s Mayoral Office, which led to almost 90 000 views by April 2023 (89860), with direct interactions of over 1500 (1679). The project’s YouTube channel, ‘COVID-19 Comunicación y Comunidad Zona Sud Cbba’ was established to disseminate co-created videos from the ECO Project. Up to April 2023, the videos registered 2473 views. The reach of WhatsApp was not possible to assess because of the informal sharing of videos and other materials. Three radio channels played the radio advertisements/drama a total of 632 times over a period of two months. During the campaign, the ECO-focused COVID-19 health talks at the participating facilities were presented a total of 79 times.

#### Qualitative data collection

The focus groups with health care personnel revealed a preference for printed material (such as posters and leaflets) to use in facilities and in community meetings. This contrasted with the importance of social media for the community, and particularly for the co-creation participants. The focus groups with health care staff and community members also highlighted persistent challenges in communicating health information effectively to the community. Despite the efforts of the education and information campaign, these included the need for medical information using non-technical language. Focus group participants also identified how they needed more information about the impacts of the COVID-19 on the body, including after infection, about the treatments on offer, and about preventing infection. The participants highlighted how different routes of communication were needed for different population groups, particularly online tools for younger people. The need for information in Quechua and Aymara was also emphasized.

#### Questionnaire-based KAP survey

The information campaign was evaluated via a KAP survey with community members and an online survey aimed at health care personnel. The in-person questionnaire-based survey sought to examine COVID-19-related knowledge, health seeking practices (including the barriers and facilitators to access health facilities) for COVID-19 diagnosis and treatment, attitudes to COVID-19 diagnosis and treatment, attitudes to the health information campaign, and the levels of confidence with respect to diagnosis and treatment of COVID-19 in the health facilities.

To reach the target sample size of 594, the survey teams visited 1,125 homes. Of these survey responses, 591 were validated by the ECO Project team and three were excluded on quality control. Almost two-thirds (63%) of KAP survey respondents were women. The average age of survey respondents was 40 years old (ranging from 18 to 80 years of age). In terms of COVID-19-related knowledge, most respondents (88%) were aware of COVID-19, (80%) could identify the groups most vulnerable people to suffer complications from the disease, such as older adults and people with underlying diseases, and (69%) recognized that it was caused by a virus or a microbe. However, in the catchment areas of three health facilities (Gloria Alto Buena Vista, Villa Israel, and K’ara K’ara), between 28% and 25% of the respondents surveyed reported being unaware of COVID-19. Overall, 60% of survey respondents reported that they or family members had previously had COVID-19. Over half (54%) reported that they would visit the health center in case of a suspected COVID-19 infection.

When responding to the question, ‘How much do you know about COVID-19?’, 28% indicated that they know enough, and 35% answered that they know the basics. Just under two-thirds of respondents (65%) recognized that they needed more information on COVID-19. Just over one in five (21%) of those surveyed had seen or heard some material from the information campaign, of which 63% recalled a key message, such as ‘go to your health center’, ‘do it on time’, or ‘COVID has not gone away’.

#### Healthcare worker online survey

A total of 95 members of staff at the participating health facilities responded to the online questionnaire. These included 35 physicians, 46 nurses and 14 laboratory staff members from across all 11 participating health facilities, of which 32 were employed at the referral hospital. Just over half (51%) reported being aware of the ECO Project and its activities. Of these respondents, 92% described material donated by the project to the health facility, with rapid diagnostic tests the most frequently recognized contribution (reported by 28 respondents). In terms of the information and education campaign, when asked about the impact of the campaign on the treatment seeking of the catchment population, more than two-thirds (69%) of those who were familiar with the ECO project, described it having a positive or very positive impact. In terms of the campaign materials, the respondents reported that the banners at the health centers were the most useful (29%) followed by the information leaflets (25%).

## Discussion

Qualitative and quantitative data collected alongside the community engagement program for the ECO Project in Cochabamba, Bolivia, highlighted the value of a multi-phase approach, with an initial formative assessment on which subsequent activities could be adapted. Moreover, in this first phase, it became apparent that, because there had been a lack of communication with the wider community from health staff, that the engagement – and particularly the information and education campaign – was responding to a clear need. The results of the questionnaire-based survey with health personnel and the qualitative data indicated a positive response to the engagement activities, the KAP survey indicated the need for longer term engagement and to strengthen further COVID-19-related knowledge, attitudes and practices. Several key lessons for community engagement in infectious disease outbreaks and epidemics were identified and are described below.

### Early engagement of community leaders

In other infectious disease studies and programmes, the early involvement of diverse stakeholder groups has been identified as key to effective community engagement [24]. Involving community leaders in the initial phases of the ECO Project was key: it enabled the project to identify those groups who were indispensable, particularly the community organizations linked to the health facilities. Community leaders facilitated the recruitment of participants for co-creation, which was foundational to the information and education campaign and to implementation of the project. This initial formative phase of the engagement (combined with the ongoing community listening) had additional benefits for the ECO Project. Indeed, connecting the community engagement into the wider project meant that the results of the formative assessment could inform the project’s efforts to support for COVID-19 diagnosis and treatment in facilities such that they were based on the needs and preferences of health staff. This highlights the benefit of integrating community engagement into wider project activities, with regular communication between the engagement and wider project team essential.

### Community listening for responsive engagement

The approach of continuous community listening helped the ECO Project to deal with changing circumstances during the pandemic, for example, as attitudes and behaviors changed among the target population. A clear example was the changes in circulating misinformation, to which the project could respond by adjusting the communication campaign. The continuous listening also enabled a response to health system needs, particularly the lack of rapid tests and well-ventilated spaces for the taking of nasal swabs. The project also had to manage the epidemiological uncertainty, which complicated the project initiation and planning of activities throughout its duration. The need for flexibility and a rapid response was clear. However, this contrasted with the time needed to develop meaningful relationships necessary for effective engagement with the community [25]. This highlights the need to maintain capacity for engagement – potentially through strengthening primary care or community health workers – outside of health emergencies [2].

### Addressing COVID-19-related mis-information with responsive community engagement

During the formative assessment and community listening, diverse but interlinked forms of misinformation on- and offline were identified as relevant to COVID-19-related attitudes and behaviors. With similar denialist responses or ‘conspiracy theories’ present across many contexts during the pandemic [26,27], it was certain that they would be encountered in Zona Sur. The ongoing community listening enabled the project to adapt engagement, particularly the information and education campaign, to respond to the fluid streams of misinformation encountered on- and offline by the community members. Given their social appeal and their links to a generalized lack of trust in state authority and (medical) expertise, it is also unsurprising that such messages are pervasive and can withstand challenge from information and education campaigns [28]. There were pockets of respondents to the KAP survey around specific health facilities that suggested that, at least in those areas, denialism remained among a minority after the information and education campaign. Although the results indicate confidence in the ECO project, but without addressing the crisis of trust in institutions and the state that often provides an environment for misinformation to flourish, such stories are likely to persist.

### Collaborative implementation of community engagement

Directly involving community members as active participants in community engagement has been identified as an important element of its success [24]. The ECO Project hence involved members of the target population in the co-design sessions and also in the administration of the KAP survey in the communities. For the co-design sessions, appealing to young people with an interest in social media and in developing their skills, meant that this was an engaged group. Offering training and providing a certificate to acknowledge their contribution and skills was key also to maintaining their active involvement. Involving community members in the KAP survey facilitated access to the communities and likely increased the response rate. The local health committees informed their communities about the upcoming KAP survey and accompanied the community health researchers as guides to the informal settlements, to ensure their security and to increase the response rate.

### Importance of well-designed monitoring and evaluation of community engagement

In terms of monitoring the community engagement activities and assessing their impact, particularly the information and education campaign, it was clear that representatives of the community groups and the health facilities participated in disseminating the material. Because of the ways in which videos, images and messages were shared online and in messenger groups, the views on social media platforms are very likely an underestimate and presented a challenge for assessing the reach of the campaign. It was a challenge also to keep track of the large amount of printed material distributed to individuals – the ECO Project was only able to record what was delivered to and activities undertaken at specific health facilities. Most tellingly, the absence of a control (non-intervention) group or a baseline assessment made it difficult to attribute causal relationships between the COVID-19-related KAPs survey and the information and education campaign.

There were two specific issues regarding the assessment of COVID-19 knowledge, attitudes and practices in the survey. First, responses to the KAP survey were also likely influenced by language issues, particularly for respondents who were more comfortable using Quechua. Lower levels of awareness about COVID-19 among people who communicate more readily in Quechua (than Spanish) have also been recorded in Peru [29]. It is possible that the 12% of survey respondents who indicated that they did not know about COVID-19 may have done so because they did not want to be associated with a stigmatized disease. There are also linguistic issues, with the verb ‘to know’ in Quechua has connotations of lived experience. Hence, of the 7% of respondents who responded in Quechua, 27% indicated that they do not ‘know’ about COVID. In addition, in the three areas (Gloria Alto Buena Vista, Villa Israel, and K’ara K’ara) where a greater proportion of respondents were unaware of COVID-19, the team identified the influence of groups who denied COVID-19 in the catchment areas. These findings emphasize the crucial role of careful translation of information and education campaigns into all languages spoken by the target communities. They also highlight the need for careful translation of questionnaires that assess the impact of these messages.

In the KAP survey, to the question, ‘have you or anyone in your family had COVID?’, 60% answered yes, and 40% no. The high proportion that responded no to this question might have resulted from the stigmatized nature of the disease, or lack of a clear confirming diagnosis. The stigmatized nature of COVID-19 infection was also potentially a factor in response to the questions about awareness and experiences of COVID-19 posed in the survey. Stigma around COVID-19 has been identified in many contexts and has been linked to fears of contagion (combined with policies of mandated quarantine and isolation), misinformation, and discrimination against particular groups perceived to experience high infection rates [30,31]. This also further highlights the need for effective information and education campaigns that are locally owned and involve leadership representatives.

### Strengths and limitations

Throughout the community engagement in the ECO project, a variety of data collection techniques were used to adapt the activities in a continuous manner and to generate broader lessons. The data were collected during formal interactions, such as focus groups and questionnaire-based surveys, and during project meetings, and also more informal interactions, such as at facility site visits. Because of the overall project design, which emphasized scaling up test and treat during a global health emergency, a baseline survey was not undertaken (nor was there a control group in a classically controlled randomized approach). Assessing precisely the impact of the community engagement, in terms of the intended outcomes, was not possible and this limits the conclusions that can be drawn.

## Conclusion

The community engagement program for the ECO Project took a multi-stage approach, with an initial formative assessment on which subsequent activities including intervention in the wider ECO project, could be adapted. This first phase highlighted a previous lack of communication between health staff and the wider community concerning access to COVID-19 diagnosis and treatment, lack of general information and misinformation about COVID-19. The engagement program and particularly the information and education campaign hence responded to a clear need. The role of key community stakeholders became clear and they were involved throughout the stages of the project. Other community members, in particular young people, took on active roles in needs identification, cocreating messages and implementing the campaign. The step-wise approach complemented by ongoing community listening enabled the creation of spaces of dialogue between community leaders, community members, health personnel and health authorities, which ultimately strengthened their relationships.

## Supplementary Material

ECO Bol supp info 1.docx
