# Supplementary material for: Lessons from community engagement to improve COVID-19 diagnosis and treatment in Cochabamba, Bolivia
Source: Glob Health Action. 2024 Jun 11;17(1):2358602. doi: 10.1080/16549716.2024.2358602 (PMC11168335; doi:10.1080/16549716.2024.2358602)
Supplement: ECO Bol supp info 1.docx [file ZGHA_A_2358602_SM8734.docx]

Table 1. The theory of change for community engagement in the ECO Project

| Outcomes | Indicators | Means of verification | Assumptions |
| --- | --- | --- | --- |
| Generate information on the behavior of the population in relation to the diagnosis and treatment of COVID-19 | Identify the health seeking routes with regard to COVID-19 diagnosis and treatment | KAP survey | The survey protocol is validated  Valid informed consent  Local authorities / leaders of social organizations have facilitated access to communities |
|  | Perceptions about barriers to accessing COVID-19 diagnosis and treatment in health facilities |  |  |
|  | Perceptions about facilitators to accessing COVID-19 diagnosis and treatment in health facilities |  |  |
| Strengthen the knowledge, attitudes and practices of the population to achieve early diagnosis and treatment of COVID-19 (within the first 5 days) particularly in populations with risk factors | Percentage of the population aware of the importance of early diagnosis and treatment (within 5 days) |  | Approval and support of local health authorities, health personnel and the leaders of social organizations to carry out the information campaign |
|  | Percentage of the population with a positive attitude toward the early diagnosis and treatment of COVID-19 |  |  |
|  | Percentage of positive social media reactions to the information campaign | KAP survey  Social media metrics  Use of paper-based material in facilities |  |
| Assess the acceptability and feasibility of a diagnostic and treatment strategy from the perspective of the community and health personnel. | Users’ level of confidence in the health facility regarding timely diagnosis and treatment of COVID-19 | KAP survey  Community focus groups | Diagnosis and treatment strategy implemented |
|  | Health personnel’s level of confidence in the health facility regarding timely diagnosis and treatment of COVID-19 | Survey with health personnel  Health personnel focus groups |  |
